# Adherence to prescribed artemisinin-based combination therapy in Garissa and Bunyala districts, Kenya

**DOI:** 10.1186/1475-2875-10-281

**Published:** 2011-09-23

**Authors:** Harriet Lawford, Dejan Zurovac, Laura O'Reilly, Sarah Hoibak, Alice Cowley, Stephen Munga, John Vulule, Elizabeth Juma, Robert W Snow, Richard Allan

**Affiliations:** 1 The MENTOR Initiative, La Prade, 11150, Villasavary, France; 2 Malaria Public Health and Epidemiology Group, Centre for Geographic Medicine, (KEMRI) - Univ. Oxford - Wellcome Trust Collaborative Programme, Nairobi, Kenya; 3 Centre for Tropical Medicine, Nuffield Department of Clinical Medicine, University of Oxford, CCVTM, Oxford, UK; 4 Centre for Global Health and Development, Boston University, Boston, MA, USA; 5 Kenya Medical Research Institute, Centre for Global Health Research, Nairobi, Kenya; 6 Department of Malaria Control (DOMC), Ministry of Public Health and Sanitation, Nairobi, Kenya

## Abstract

**Background:**

Following the development of resistance to anti-malarial mono-therapies, malaria endemic countries in Africa now use artemisinin-based combination therapy (ACT) as recommended first-line treatment for uncomplicated malaria. Patients' adherence to ACT is an important factor to ensure treatment efficacy, as well as to reduce the likelihood of parasite resistance to these drugs. This study reports adherence to a specific ACT, artemether-lumefantrine (AL), under conditions of routine clinical practice in Kenya.

**Method:**

The study was undertaken in Garissa and Bunyala districts among outpatients of five government health facilities. Patients treated with AL were visited at home four days after having been prescribed the drug. Respondents (patients ≥ 15 years and caregivers of patients < 15 years) were interviewed using a standardized questionnaire, AL blister packs were physically inspected and the adherence status of patients was then recorded. Multivariate logistic regression modelling was used to determine predictors of adherence.

**Results:**

Of the 918 patients included in the study, 588 (64.1%) were 'probably adherent', 291 (31.7%) were 'definitely non-adherent' and 39 (4.2%) were 'probably non-adherent'. Six factors were found to be significant predictors of adherence: patient knowledge of the ACT dosing regimen (odds ratio (OR) = 1.76; 95% CI = 1.32-2.35), patient age (OR = 1.65; 95% CI = 1.02-1.85), respondent age (OR = 1.37; 95% CI = 1.10-2.48), whether a respondent had seen AL before (OR = 1.46; 95% CI = 1.08-1.98), whether a patient had reported dislikes to AL (OR = 0.62 95% CI = 0.47-0.82) and whether a respondent had waited more than 24 hours to seek treatment (OR = 0.73; 95% CI = 0.54-0.99).

**Conclusion:**

Overall, adherence to AL was found to be low in both Garissa and Bunyala districts, with patient knowledge of the AL dosing regimen found to be the strongest predictor of adherence. Interventions aimed at increasing community awareness of the AL dosing regimen, use of child friendly formulations and improving health workers' prescribing practices are likely to ensure higher adherence to AL and eventual treatment success.

## Background

*Plasmodium falciparum *causes approximately 0.5 billion clinical episodes each year and the majority are borne by populations living in Africa [1,2]. The last 10 years has witnessed a rapid scaling-up of malaria prevention due to massive increases in donor assistance to support malaria control in Africa [3]. The greatest threat to the future of malaria control during the early part of this decade was the rapid and wide-spread emergence of resistance to widely used mono-therapies, such as chloroquine (CQ) and sulphadoxine/sulphalene-pyrimethamine (SP) [4,5]. However, between 2003 and 2010, all malaria endemic countries in Africa switched to using artemisinin-based combination therapy (ACT) as a recommended first-line treatment for uncomplicated malaria.

Artemisinin-derived drugs are fast acting anti-malarials with high clinical efficacies and rapid parasite elimination profiles that, when combined with other compounds with different parasite targets, are likely to reduce the probability and speed of resistance [6]. However, most ACT preparations have more complex dosing regimens than previously used mono-therapies [7]. Recent research has focussed on factors that influence a patient's adherence to the full course of ACT, including dosing complexity, costs and perceived rapid improvement, and how they can impact on clinical and pharmacological efficacy [8-15]. In theory, approaches that reduce patient non-adherence with ACT should mitigate the risks of resistance emerging in areas of high parasite transmission. There is a growing need to address the possibilities of reduced efficacy to artemisinin in Africa following alarming reports of resistance and treatment failure to both artemisinin-mefloquine and artemether-lumefantrine (AL) combinations along the Cambodia-Thailand border and in Vietnam [16-18]. Given that resistance to SP and CQ are believed to have originated from similar foci in South-Eastern Asia [19-21], concerns have been raised that resistance to ACT may spread to Africa if resistance is not contained and ACT use in Africa is not regulated [22,23].

In Kenya, first-line treatment recommendations for uncomplicated malaria were changed from CQ to SP in 1998, and then from SP to AL in 2004; however this policy change was not implemented until the second part of 2006 when AL was distributed to government and mission public sector facilities, revised national standard treatment guidelines were changed and front-line health workers underwent in-service training [24]. However, since 2006 there have been problems associated with drug procurement and supply [25], coverage of health worker training and supervision [26-28] and clarity on how to prescribe AL [7]. This was an observational study of patient adherence to AL under standard conditions of clinical practice in two regions of Kenya.

## Methods

### Study sites

The study was undertaken in two districts: Bunyala district, Western Province, close to Lake Victoria with perennial malaria transmission and community-based parasite prevalence among children in excess of 40% [29], and Garissa district, North-Eastern Province, a semi-arid region with acutely seasonal malaria transmission and childhood parasite prevalence of less than 1% during the dry season [29]. Two government health facilities were selected in Bunyala district (Budalangi dispensary and Mukhobola health centre), and three dispensaries in Garissa district (G. K. Prison, Utawala and Young Muslim). Mukhobola had an outpatient, as well as an inpatient facility, whilst the remaining four had outpatient facilities only. At outpatient facilities, the clinical officers or community health nurses were responsible for prescribing and dispensing AL.

### Study population

Patients were recruited from selected health facilities between September and December 2009 in Bunyala, and between July and August 2010 in Garissa. Inclusion criteria were: testing positive for malaria parasites using slide microscopy or rapid diagnostic tests, having a weight of ≥ 5 kg and being more than 5 months of age. If these patients had reported no other anti-malarial use during the preceding two weeks, had no signs of complicated malaria or other severe disease, and did not have another household member participating in the study, they were recruited until 646 patients had been enrolled in Budalangi in three age groups (< 5 years, 5-14 years and ≥ 15 years), and 272 had been enrolled in Garissa in two age groups (< 14 years and ≥ 15 years). Given the low transmission of malaria during the dry season in Garissa, the sample size had been reduced to accommodate for fewer patients attending the health facilities. Correspondingly, sample size was based on a projected adherence of 70% in Garissa and 60% in Budalangi, 10% precision in the point estimate, a 5% type-1 error and an estimated 20% loss to follow-up (EpiInfo 3.5.1, CDC, WHO).

### Study procedures

All patients that fulfilled the entry criteria and were prescribed AL were asked to keep their blister packs once they had completed the medication, and details were recorded of each patient's residential address and clinic card. Patients were not told why their details were being taken or why they were required to keep their blister packs, thus ensuring patient adherence was not affected by knowledge of the survey.

On the fourth day after recruitment, patients were followed-up at home, by which time all doses should have been taken. All respondents, in this case patients aged 15 years or more and caregivers of patients aged less than 15 years, had the study explained to them in a language they were most familiar with, and written informed consent was obtained. Interviews were conducted by field officers using a structured questionnaire written in English and translated into KiSomali (Garissa district), BaLuya (Bunyala district) or KiSwahili where appropriate (see Additional File 1). The questionnaire included information on socio-demographic characteristics of the patient/caregiver and of their household (household size, education level, employment status), details of information provided by the prescribers at the health facilities (whether or not they were told how to take AL correctly), respondent knowledge of the AL dosing regimen and the patient's acceptance of the AL treatment (whether or not any dislikes or side-effects were reported). Respondent knowledge regarding the ACT dosing regimen was assessed by asking respondents directly how AL should be taken, and the number of correct statements given was totalled. Correct statements included taking the first dose immediately, taking the second dose after 8 hours, taking the remaining doses in the morning and evening, taking doses with milk/fatty foods, and taking all the tablets. Additionally, blister packs were directly observed and the patient's adherence status was then recorded. Patients identified on the day of follow-up who had not completed the course were asked why, and were then advised to return to a health facility for follow up treatment.

### Analysis

Open-ended questions were reviewed and grouped into thematic categories that were coded for data entry. Results were double entered into EpiInfo 3.5.1 (CDC, WHO) and all data were analysed in STATA 11.0 (Stata Corporation, College Station, Texas). Patients who had a blister pack still containing ACT when interviewed were considered 'definitely non-adherent', and patients were categorized as 'probably non-adherent' if they or their caregivers claimed the doses were not completed but did not show a blister pack. 'Probably adherent' patients were those who claimed to have completed all the doses and could show and empty blister pack, as well as patients who could not show a blister pack, but claimed to have taken all the tablets.

To identify predictors related to adherence, data from the two study sites were combined and chi-square tests were first performed in a univariate logistic regression model. Outcomes were grouped by those considered 'probably adherent' and those considered either 'probably' or 'definitely non-adherent'. Factors tested at univariate logistic regression included demographic information relating to the respondent, patient and head of household, as well as health-seeking behaviour and patient acceptance of AL. To increase statistical power and simplify interpretations, multilevel categorical variables were collapsed into binary ones. A forward stepwise selection procedure was performed. Predictors found to be significant at the *P *≤ 0.25 level were entered into a multivariate logistic regression model with remaining non-significant predictors introduced one at a time to detect for additional confounders. These remained in the model if the OR of other predictors in the model changed by greater than 20%, though no additional confounders were found. A backwards stepwise elimination procedure was performed with a *P *≤ 0.05 criterion for remaining in the model. All regression analyses were undertaken adjusting for clustering at health facility level. Any questionnaires with missing data were not included in the regression models.

### Ethical considerations

Ethical approval for the study was provided by the Kenya Medical Research Institute/National Ethical Review Committee (protocol number 1742).

## Results

### Description of population

A total of 893 patients in Bunyala district were enrolled; 87 (9.7%) were excluded from interview because members of their household had already been interviewed, 80 (9.0%) refused participation and 80 (9.0%) were lost to subsequent follow-up. Of the remaining 646 patients, approximately equivalent recruitment was obtained across the three age groups and gender. A total of 351 patients were enrolled in Garissa. Of these, 43 (12.3%) did not meet the inclusion criteria as a member of their household had already been interviewed, two (0.6%) refused to participate and 34 (9.7%) were lost to follow-up and thus were not included in the final analysis. Approximately equivalent age group recruitment was observed in the final sample of 272 patients, though these groups were significantly different regarding sex distribution. The characteristics of the recruited study populations in both districts are shown in Table 1.

**Table 1:** Main characteristics of study participants

Bunyala District	< 5		5 - 14		≥ 15		Total	
	n = 212		n = 213		n = 221		n = 646	
Patient sex*								
Male	99	(47.1%)	96	(45.1%)	89	(40.3%)	284	(44.2%)
Female	111	(52.9%)	117	(54.9%)	130	(59.4%)	358	(55.8%)
Education of head of household*								
Never attended	13	(6.5%)	12	(6.1%)	23	(11.3%)	48	(8.0%)
Primary	130	(65.0%)	97	(49.2%)	95	(46.8%)	322	(53.8%)
Secondary	45	(22.5%)	60	(30.5%)	53	(26.1%)	158	(26.4%)
Above secondary	10	(5.0%)	28	(14.2%)	32	(15.8%)	70	(11.7%)
N/A	2	(1.0%)	0	(0.0%)	0	(0.0%)	2	(0.3%)
Employment of head of household*								
Agriculture	81	(40.3%)	83	(41.9%)	87	(43.1%)	251	(41.8%)
Trade/private business	38	(18.9%)	36	(18.2%)	41	(20.3%)	115	(19.1%)
Fishing	54	(26.9%)	34	(17.2%)	25	(12.4%)	113	(18.8%)
School	1	(0.5%)	1	(0.5%)	0	(0.0%)	8	(4.0%)
Unemployed	9	(4.5%)	11	(5.6%)	2	(1.0%)	22	(3.7%)
Household size*								
0-5	124	(58.5%)	80	(38.1%)	116	(57.4%)	320	(51.3%)
≥ 6	88	(41.5%)	130	(61.9%)	86	(42.6%)	304	(48.7%)
Children < 5 years in household*								
≤ 1	65	(30.7%)	139	(66.2%)	146	(72. 3%)	350	(56.1%)
> 1	147	(69.3%)	71	(33.8%)	56	(27.7%)	274	(43.9%)

**Garissa District**			**< 15**		**≥ 15**		**Total**	
			**n = 136**		**n = 136**		**n = 272**	

**Patient sex**								
Male			71	(52.2%)	41	(30.2%)	112	(41.2%)
Female			65	(47.8%)	95	(69.9%)	160	(58.8%)
**Education of head of household**								
Never attended			94	(69.1%)	84	(61.8%)	178	(65.4%)
Primary			16	(11.8%)	22	(16.2%)	38	(14.0%)
Secondary and above			26	(19.1%)	30	(22.1%)	56	(20.6%)
**Employment of head of household**								
Farming/other			39	(30.2%)	51	(37.5%)	90	(33.1%)
Housewife			18	(13.2%)	7	(5.2%)	25	(9.2%)
Own business			35	(25.7%)	39	(28.7%)	74	(27.2%)
Retired/unemployed			43	(31.6%)	38	(27.9%)	81	(29.8%)
In school			1	(0.7%)	1	(0.7%)	2	(0.7%)
Household size*								
0-5			36	(26.5%)	47	(34.8%)	83	(30.6%)
≥ 6			100	(73.5%)	88	(65.2%)	188	(69.3%)
Children < 5 years in household*								
≤ 1			57	(41.9%)	80	(59.3%)	137	(50.6%)
> 1			79	(58.1%)	55	(40.7%)	134	(49.5%)

*Not all households provided complete demographic information in the survey

### Adherence - Bunyala and Garissa district

Of the 918 patients treated with AL, 697 (76.1%) retained and showed a treatment blister pack during the home visit. Overall, 588 (64.1%) patients claimed to have taken all tablets and were considered 'probably adherent', of whom 69.2% (407/588) showed empty blister packs during the home visit. A further 291 (31.7%) patients had AL doses remaining in their blister pack and were therefore considered 'definitely non-adherent'. Only 39 (4.3%) patients reported they had taken their drugs incorrectly and had not retained their blister packs. These patients were therefore categorized as 'probably non-adherent'. Nearly equal proportions of adherence were observed in the two study sites (Table 2).

**Table 2:** Patients' adherence to artemether-lumefantrine in study districts

	Bunyala		Garissa	
	n = 646		n = 272	
Probably adherent	412	(63.8%)	176	(64.7%)
Definitely non-adherent	207	(32.0%)	84	(30.9%)
Probably non-adherent	27	(4.2%)	12	(4.4%)

Univariate logistic regression analysis compared 19 predictors of patients who were classified as adherent (n = 588) versus those that were not adherent (n = 330). Fourteen predictors with *P*-value ≤ 0.25 were fitted in a multivariate logistic regression model: patient age (< 15 and ≥ 15 years); respondent and head of household age (< 25, 25-50 or > 50 years); respondent education level (never attended, primary level or secondary level and above); if the respondent and head of household was employed; household size (0-5 and ≥ 6); if patient had waited more than 24 hours to seek treatment; if respondent had taken AL before; if respondent had seen AL before, if patient had any dislikes or side-effects to the treatment, patient knowledge (no knowledge of how to take AL and at least one correct statement) and if the patient had been given any instructions as to how to take AL at the health facility. Six variables were significantly (*P *< 0.05) associated with the outcome after allowing for potential confounders (Table 3). The strongest predictor of adherence concerned patient knowledge of the ACT regimen; if a patient was able to cite at least one correct instruction as to how to take AL, they had 1.76 (95% CI = 1.32-2.35) the odds of being fully adherent than a respondent () that could cite none. Other significant factors included whether a respondent was aged between 25-50 years as opposed to less than 25 years (odds ratio (OR) = 1.65, 95% CI = 1.10-2.48); whether a patient was aged 15 years or more as opposed to less than 15 (OR = 1.37, 95% CI = 1.02-1.85); whether a respondent had seen the drug before or not (OR = 1.46, 95% CI = 1.08-1.98); whether a patient had reported dislikes to medication or not (OR = 0.62, 95% CI = 0.47-0.82) and if a respondent had waited more than 24 hours to seek treatment as opposed to seeking treatment in ≤ 24 hours after becoming symptomatic (OR = 0.73, 95% CI = 0.54-0.99).

**Table 3:** Bivariate (crude) and multivariate (adjusted) model of association between adherence and exposure variables

Variable	No. of patients	Adherent		Crude OR		*P *Value	Adjusted OR		*P *Value
	n = 918	n = 588 (%)		(95% CI)			(95% CI)		
**Patient age**									
< 15	561	346	(61.7)	1.0 (ref)					
≥ 15	357	242	(67.8)	1.31	(0.96-1.78)	0.087	1.37	(1.02-1.85)	0.038
**Respondent age**									
13-24	284	157	(55.3)	1.0 (ref)					
25-50	546	376	(68.9)	1.79	(1.42-2.25)	< 0.0001	1.30	(1.10-2.48)	0.016
≥ 51	80	49	(61.3)	1.28	(0.84-1.95)	0.256	1.58	(0.63-2.45)	0.533
**Days waited to attend health facility**									
≤ 1	453	310	(68.4)	1.0 (ref)					
> 1	465	278	(59.8)	0.69	(0.55-0.85)	0.001	0.73	(0.54-0.99)	0.046
**Respondent seen drug before**									
No	123	73	(59.3)	1.0 (ref)					
Yes	791	513	(64.9)	1.26	(0.87-1.85)	0.225	1.46	(1.08-1.98)	0.014
**Dislikes**									
No	811	533	(65.7)	1.0 (ref)					
Yes	107	55	(51.4)	0.55	(0.35-0.87)	0.011	0.62	(0.47-0.82)	0.001
**Patient number of correct statements**									
No correct statements	170	87	(51.2)	1.0 (ref)					
At least one correct statement	748	501	(67.0)	1.94	(1.49-2.51)	< 0.0001	1.76	(1.32-2.35)	< 0.0001

## Discussion

Adherence to AL is of particular importance given the large scale-up of ACTs in sub-Saharan Africa [30]. This study has revealed that patient adherence to AL, four years after the national roll-out of the new treatment policy in Kenya, was 64%. Overall, adherence levels were found to be considerably lower in this study than in other similar studies. Only 64% of patients from Bunyala and Garissa district were 'probably adherent' and had taken the full six-dose regimen, whereas 98% of patients in a study in Tanzania were reported to be 'probably adherent' one year after AL was adopted as a first-line treatment for uncomplicated malaria [11], and 90% adherence to the same drug was reported in Uganda [9]. A potential reason for the discrepancies in adherence levels between these studies is due to the operational conditions under which they were conducted; in Tanzania and Uganda, health workers had been trained in the use of anti-malarials prior to the study, the first AL dose was administered at facilities under the direct observation, and adequate counselling on how to take AL was systematically provided [31]. However, as this study was carried out without the involvement of study teams in the treatment of patients, it may be a more accurate reflection of true adherence to AL under normal, real-life conditions. Similarly, Beer *et al *[31] and Depoortere *et al *[32] investigated adherence under routine conditions and found 77% and 39% adherence, respectively, with both studies attributing poor adherence to weaknesses at prescription level. Likewise, this study highlights poor patient knowledge of the AL dosing regimen as a reason for low adherence, a factor that can also be improved with better prescribing practice at health facilities.

Correct knowledge of the AL dosing regimen is critically important as non-adherence can lead to treatment failure, recrudescence and increase the risk of malaria parasite resistance [18]. For optimal results, the first dose of AL must be taken immediately to ensure high levels of artemether in the blood, followed by the second dose after a strict 8-hour interval to maintain a high level of the drug in the bloodstream [9,33]. After introducing health worker counselling of patients in Nigeria and Tanzania, adherence was found to reach 73.3% and 75.0%, respectively [34,35]; this indicates that improving prescriber practice and counselling of patients during health facility visits is likely to increase patient knowledge and improve adherence. This study also found a significant association between patient knowledge and adherence; patients who could give at least one correct answer as to how to take AL had 1.76 the odds of taking all the tablets. The issue of education on the subject of the AL dosing regimen is a major predictor of adherence and focus on improving instructions given to patients and caregivers at health facilities, and thereby increasing caregivers' and patients' understanding of the treatment, could help to improve treatment with AL.

The relative bioavailability of artemether is known to increase more than two-fold, and that of lumefantrine sixteen-fold, when AL is taken after a high-fat meal, compared to fasted conditions [33]. In this study, only 41.4% of patients receiving AL knew to take this with food or milk. Encouraging health workers to correctly educate patients to take AL with fatty foods may help improve the curative efficacy of the treatment and subsequently the confidence of communities using AL.

Respondent age was a significant predictor for full adherence to AL in this study. A similar association was reported in Senegal, where it was concluded that older caregivers spent more time at home, could better attend to younger patients and knew more with regards to administering ACT [14]. It is likely that older respondents may have had prior experience administering or taking AL, which is supported by evidence from this study that indicates respondents who had seen AL before, had the odds 1.46 of adhering fully.

Patient age was also an important predictor of adherence; children aged 15 years or over, and adults, who receive AL are more likely to be adherent than children aged less than 15. This implies that younger children may need more help remembering to take anti-malarials and ensuring that all doses are completed. Conversely, in Senegal, children aged 8 to 10 were three times more likely to be non-adherent than children 2 to 4 years. In this case, children aged 8 to 10 were reported to have more responsibilities and be less supervised by caregivers, and were more likely to either forget to take doses, or stop taking doses as soon as they felt better [13]. Encouraging health workers to reinforce the importance of the caregiver's role and ensure their understanding of administering treatment may help to improve adherence in children.

This study indicates that early treatment seeking for AL was positively associated with improved adherence; patients who took more than 24 hours to seek treatment at a health facility were 27% less likely to adhere fully. Late treatment seeking for malaria is associated with increased mortality as most deaths occur within 24-48 hours of onset of symptoms [36]. The importance of encouraging caregivers and patients to seek early diagnosis and effective treatment is vital if malaria mortality rates are to be successfully reduced. In this study 51% of malaria patients delayed more than 24 hours before seeking treatment.

Disadvantages of AL include the large number of tablets that need to be taken to complete the full course of therapy. This is further complicated by the fact that the first two doses of AL need to be taken 8 hours apart, and the remaining doses taken twice a day in the morning and evening. In this study, patients reporting dislikes to AL, such as taste and smell, were less likely to be fully adherent (OR = 0.62). Increasing the acceptability of AL is very important in Kenya, where it was found 36% of people are non-compliant. Other studies have focussed on addressing dislikes to AL in children, with a recent study in collaboration with Novartis^® ^evaluating a novel dispersible tablet of Coartem^® ^in different flavours. These are specifically designed with clear instructions to promote adherence and the sweeter flavour encourages children to take their medication [37]. Increased use of dispersible AL may reduce difficulties administering medication to children, and therefore positively improve adherence in the future. Manufacturers of other formulations of ACT have addressed acceptability and adherence issues by significantly reducing the daily number of tablets required [38].

Given that the reported associations between predictors and adherence to AL in this study are weak (OR < 2), this may also indicate that the true causes of adherence were not identified and that further investigation using different experimental designs need to be explored. This study has been valuable in identifying a method to quantitatively determine adherence. However, to fully understand the factors that cause adherence to AL, further qualitative studies should be conducted using methods such as focus group discussions and monitoring of consultations at health facilities. Patient knowledge of the dosing regimen has been shown to be associated to adherence, however the role of patient knowledge of the AL dosing regimen, patient awareness of malaria and adherence to anti-malarials should be investigated further to determine if they are true predictors of adherence, both at an individual and a community level.

## Conclusion

Overall, adherence to AL in Garissa and Bunyala districts was low. This study has highlighted that improving patient knowledge regarding the AL dosing regimen may improve the likelihood of patients adhering fully to AL. Focusing on health worker training and prescribing practice is essential; materials such as posters and videotapes have been seen to improve health worker performance [32,39] and the distribution of treatment guidelines and posters are a good short-term solution. Targeted information, education and communication activities at the community level may help to increase awareness of the AL treatment regimen and its uptake, and reduce the risk of contributing to the development of parasite resistance. Individuals should be encouraged to seek health care as soon as possible within 24 hours of becoming symptomatic. This may reduce the risk of severe malaria infection and mortality and also improve adherence to medication.

## Competing interests

The authors declare that they have no competing interests.

## Authors' contributions

RA, SH and AC conceptualized and designed the study. HL was the principal investigator, and HL, LOR and AC were responsible for the implementation and supervision of the study. SM, JV and EJ provided technical support. HL, DZ, RWS and LOR analysed and interpreted the study. HL, DZ, RWS and RA wrote the manuscript. All authors read the final manuscript and approved.

## Supplementary Material

Additional file 1 **ACT adherence survey: consent form and questionnaire**. The questionnaire and consent form used for the ACT adherence surveys to interview patients who had received ACT from a health centre four days earlier. Click here for file
