# Supplementary material for: Adherence to prescribed artemisinin-based combination therapy in Garissa and Bunyala districts, Kenya
Source: Malar J. 2011 Sep 23;10:281. doi: 10.1186/1475-2875-10-281 (PMC3189920; doi:10.1186/1475-2875-10-281)
Supplement: Additional file 1 — ACT adherence survey: consent form and questionnaire. The questionnaire and consent form used for the ACT adherence surveys to interview patients who had received ACT from a health centre four days earlier. [file 1475-2875-10-281-S1.DOC]

**Additional File 1 – Survey consent form & questionnaire**

**
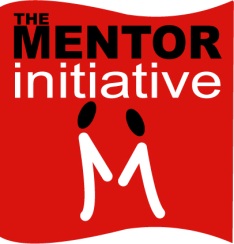
**

| **IDENTIFICATION** |
| --- |

**1. To be completed by interviewer**

**0.1**- |________________________| |__|__|

Interviewer Name Interviewer ID

**0.2**- Date: |__|__| / |__|__| / |__|__|

Day Month Year

**0.3**- Patient name ____________________________________

**0.4**- Village name ____________________________________

**0.5**- Sub location _____________________________________

**0.6**- Head of Household _______________________________

INTRODUCTION AND CONSENT

Hello. My name is and I am a representative of THE MENTOR Initiative. We are conducting a survey about malaria. The information we collect will help us plan health services. We would very much appreciate your participation in this survey.

I understand you visited the health facility recently and were given Artefan treatment for malaria. I would like to ask you information about either the child in your household or yourself who received this treatment. The survey usually takes 20 minutes to complete. Whatever answers you provide will be kept confidential and will not be shown to other persons.

We request that for children under 15 their caretaker respond to the questions with them and on their behalf.

Participation in the survey is voluntary. If you participate, you may decide to stop answering any or all questions at any time.

Even if you decide not to take part, this will have no effect on the health care or treatment you receive, and neither will any of the answers you give.

However, we hope that you will participate fully in this survey since your views are important.

At this time, do you want to ask me anything about the survey?

Do you agree to be interviewed or for your children to participate?

1……………YES 🡪 CONTINUE

0……………NO 🡪 END

Signature of Participant:

**0.7**- Household tracking:

Respondent found and consented to interview..............1

Respondent found but refused consent.........................2

Respondent not found....................................................3

Respondent not home....................................................4

– date of visit 1|__|__| / |__|__| / |__|__|

2|__|__| / |__|__| / |__|__|

3|__|__| / |__|__| / |__|__|

**0.8**- Has anyone in this household had this interview before? **□**

**0.9**- Before this time, when did you last have malaria?_________

**2. To be completed by team leader/supervisor**

Team Leader/Supervisor Code: |__|__|

Remarks:

__________________________________________________

__________________________________________________

__________________________________________________

_________________________________________________________________________________________________________________________________________________________

I confirm that the questionnaire is fully completed.

Date: |__|__| / |__|__| / |__|__|

Day Month Year

Name: |________________________________|

Signature: ______________________________

**3. To be completed by Data entry Clerk**

Data Entry Clerk 1 Data Entry Clerk 2

Date: |__|__| / |__|__| / |__|__| Date: |__|__| / |__|__| / |__|__|

Day Month Year Day Month Year

I confirm that the I confirm that the

questionnaire is correctly questionnaire is correctly

entered. entered.

Initials: _______________ Initials: _______________

| **SECTION 1  *Interviewer:* I WOULD LIKE TO ASK YOU SOME QUESTIONS ABOUT YOU AND YOUR FAMILY WHO LIVE IN THIS HOUSE.** |
| --- |

| **LINE NO.** | **USUAL RESIDENTS** | **SEX** | **AGE** | **EDUCATION** | **NUMBER OF NIGHTS PER WEEK IN HOUSE** | **DID THAT PERSON SLEEP HERE LAST NIGHT?** | **EMPLOYMENT** |
| --- | --- | --- | --- | --- | --- | --- | --- |
|  | Please give me the names of the persons who usually live in your household | Is (NAME) male or female? | How old is (NAME)?  **(for children < 1 record 0))** | What is your / thier level of education?  **00 = NEVER ATTENDED SCHOOL**  **01 = PRIMARY**  **02 = SECONDARY**  **03 = POST SECONDARY**  **04 = Other ___________**  **99=not applicable**  **(write 99 if less than 6 years)** | Please indicate the number of nights per week they sleep in the house |  | 1= Farming  2-= Fishing  3 = Own Business  4 = Retired  5 = In school  6 = Other___________  7 = Unemployed  9 = N/A (chlldren) |
| 1.1 | 1.2 | 1.3 | 1.4 | 1.5 | 1.6 | 1.7 | 1.8 |
|  |  | **M F** |  |  | Nights | **Yes No** |  |
| 01respondent |  | 1 2 |  |  |  | 1 0 |  |
| 02 patient |  | 1 2 |  |  |  | 1 0 |  |
| 03 Head of household |  | 1 2 |  |  |  | 1 0 |  |

| **No.** | **QUESTIONS AND FILTERS** | **CODING CATEGORIES** |
| --- | --- | --- |
| 1.9 | How many people are there in this household? | I__I__I people |
| 1.10 | How many households are there in your homestead (including yours)? | I__I__I households |
| 1.11 | How many children under 5 are there in this household? | I__I__I children under 5 |
| 1.12 | How many children aged 5 – 14 are there in this household? | I__I__I children aged 5 – 14 |
| 1.13 | How many children in this household attend school? | I__I__I children attend school |

| **SECTION 2 Interviewer*:* NOW I WANT TO ASK YOU SOME QUESTIONS ABOUT THE MALARIA TREATMENT (INSERT NAME OF PATIENT) RECEIVED THREE DAYS AGO FROM THE HEALTH FACILITY** |
| --- |

| **No.** | **QUESTIONS AND FILTERS** | **CODING CATEGORIES** | |
| --- | --- | --- | --- |
| 2.1 | Which health facility did you seek health care from? | 1……………Mukhobola Health Centre  2……………Budalangi Dispensary | |
| 2.2 | How many days did you wait to go to the health facility after (insert name of patient) first got sick? | I__I__I days  If you waited more than 1 day, please give reasons why: _____________________________________  _________________________________________ | |
| 2.3 | Did you seek treatment from anywhere else before you went to the health facility? | 0………….No **🡪 Skip to Q. 2.5**  1………….Yes | |
| 2.4 | Where did you go to seek other treatment?  *(Circle all that apply)* | 1……….…. Community Health worker  2………….. Traditional Healer  3………….. Private Pharmacy  4………….. Religious Healer  5………….. Herbalist  6………….. Other _________________  99.………... Don’t know | |
| 2.5 | At the health facility did (insert name of patient) receive a rapid diagnostic test for malaria?  (SHOW PICTURE) | 1……………Yes  0 …………...No **🡪 Skip to Q. 2.7**  99.………… Don’t know | |
| 2.6 | What was the result? | 1……………Positive  0 …………...Negative  99.………… Don’t know | |
| 2.7 | What medication were you prescribed for malaria?  *(Do not read out the options)* | 1.…… …….Artefan  2…………...Coartem  3……………AL (Artemether / Lumefantrine)  4…………...Other, specify__________________  99………….Don’t know | |
| 2.8 | Have you (*the respondent*) ever seen this drug before? | 1……………Yes  0 …………...No  99.………… Don’t know | |
| 2.9 | Have you *(the respondent)* ever taken this drug before? | 1……………Yes  0 …………...No  99.………… Don’t know **🡪 If patient is an adult,**  **skip to Q. 2.11** | |
| 2.10 | Has the patient ever taken this drug before?  *(If the patient is a child)* | 1……………Yes  0 …………...No  99.………… Don’t know | |
| 2.11 | Who provided you with the malaria medication? | 1…………...Doctor/ clinical officer/ nurse  2……………Clinic pharmacist  3……………Private pharmacy  4..................Other (specify)______________________  99…………..Don’t know | |
| 2.12 | How much did you have to pay for the malaria medication? | 1…………...20 Ksh  2…………...Nothing  3…………...Other, give amount________________  99………….Don’t know | |
| 2.13 | Who explained to you how to take the malaria medication? | 1…………...Doctor/ clinical officer  2……………Clinic pharmacist  3……………Private pharmacy  4……………Family member/friend  5...................Other (specify)______________________  99…………..Don’t know | |
| 2.14 | Describe to me how you are supposed to take the malaria medication  _______________________________________________________________________________________  _______________________________________________________________________________________  _______________________________________________________________________________________  _______________________________________________________________________________________  _______________________________________________________________________________________  _______________________________________________________________________________________ | | |
| 2.15 | Did you have any difficulties understanding how to take the malaria medication? | 1……………Yes  0…………...No **🡪 Skip to Q. 2.17** | |
| 2.16 | What difficulties did you have?  _______________________________________________________________________________________  _______________________________________________________________________________________  _______________________________________________________________________________________ | | |
| 2.17 | Did you have any dislikes about the malaria medication? | | 1……………Yes  0…………...No **🡪 Skip to Q. 2.19** |
| 2.18 | What dislikes did you have?  _______________________________________________________________________________________  _______________________________________________________________________________________  _______________________________________________________________________________________ | | |
| 2.19 | Did you have any side effects to the malaria medication? | 1……………Yes  0…………...No **🡪 Skip to Q. 2.21** | |
| 2.20 | What side effects did you have?  _______________________________________________________________________________________  _______________________________________________________________________________________  _______________________________________________________________________________________ | | |
| 2.21 | Can you show me the treatment package given? | 1……………Yes  0……………No | |

| **SECTION 3: TREATMENT COMPLIANCE** | | |
| --- | --- | --- |
|  | 3.1 | Looking at the blister pack, circle the blister pack they were given. If they cannot show you the blister pack, circle the one they say they were given. |
|  | 3.2 | Write the name on the packaging (if shown) ___________________________ |
|  | 3.3 | Scratch out the pills that you see are empty in the blister. |
|  | 3.4 | Ask them when each of the pills were taken and how each dose was taken. Indicate in each of the columns what day the pills were taken on (Day 1, 2, 3), if they were taken in the morning or the evening and whether they were taken with water, food, milk, crushed, whole etc... |

BLISTER B6 (green) BLISTER B12 (yellow)

| When taken:  How taken: | When taken:  How taken: | When taken:  How taken: |  | When taken:  How taken: | When taken:  How taken: | When taken:  How taken: |
| --- | --- | --- | --- | --- | --- | --- |
|  |  |  |  |  |  |  |
|  |  |  |  |  |  |  |
| When taken:  How taken: | When taken:  How taken: | When taken:  How taken: |  | When taken:  How taken: | When taken:  How taken: | When taken:  How taken: |

BLISTER B18 Blister B24 (orange)

|  | When taken:  How taken: | | When taken:  How taken: | When taken:  How taken: |  | When taken:  How taken: | When taken:  How taken: | When taken:  How taken: |
| --- | --- | --- | --- | --- | --- | --- | --- | --- |
|  |  | |  |  |  |  |  |  |
|  |  | |  |  |  |  |  |  |
|  | When taken:  How taken: | | When taken:  How taken: | When taken:  How taken: |  | When taken:  How taken: | When taken:  How taken: | When taken:  How taken: |
| 3.5 | | If they missed any doses, did not complete the treatment or took any at the wrong time ask them to explain why?  _______________________________________________________________________________________  _______________________________________________________________________________________  _______________________________________________________________________________________  ______________________________________________________________________________________  _______________________________________________________________________________________  _______________________________________________________________________________________ | | | | | | |
| 3.7 | | Finally, ask them whether they were given any of the following advice about taking their medication?  **No Yes**   1. Take first dose straight away  **□ □** 2. Second dose after 8 hours/ (one day) **□ □** 3. Morning & evening next 2 days **□ □** 4. Take with food/ milk **□ □** 5. Take all the tablets **□ □** 6. Repeat the dose if vomits within 30mins **□ □** 7. Finish the course **□ □** 8. Return to health facility if deteriorates/   symptoms not resolved in 3 days. **□ □** | | | | | | |

| COMMENTS: |
| --- |

**Thank the respondent for their participation and ask them if they have any questions.**

**If they have not completed the full course of treatment, advise them to return to the health clinic for a new assessment in case their condition has not been fully resolved.**

************** END OF QUESTIONNAIRE **************
